# Distinct Habitats Select Particular Bacterial Communities in Mangrove Sediments

**DOI:** 10.1155/2016/3435809

**Published:** 2016-02-17

**Authors:** Lidianne L. Rocha, Geórgia B. Colares, Vanessa L. R. Nogueira, Fernanda A. Paes, Vânia M. M. Melo

**Affiliations:** Laboratório de Ecologia Microbiana e Biotecnologia (LEMBiotech), Departamento de Biologia, Centro de Ciências, Universidade Federal do Ceará, Campus do Pici, Bloco 909, Avenida Mister Hull s/n, 60.455-970 Fortaleza, CE, Brazil

## Abstract

We investigated the relationship among environmental variables, composition, and structure of bacterial communities in different habitats in a mangrove located nearby to an oil exploitation area, aiming to retrieve the natural pattern of bacterial communities in this ecosystem. The T-RFLP analysis showed a high diversity of bacterial populations and an increase in the bacterial richness from habitats closer to the sea and without vegetation (S1) to habitats covered by* Avicennia schaueriana* (S2) and* Rhizophora mangle* (S3). Environmental variables in S1 and S2 were more similar than in S3; however, when comparing the bacterial compositions, S2 and S3 shared more OTUs between them, suggesting that the presence of vegetation is an important factor in shaping these bacterial communities.* In silico* analyses of the fragments revealed a high diversity of the class Gammaproteobacteria in the 3 sites, although in general they presented quite different bacterial composition, which is probably shaped by the specificities of each habitat. This study shows that microhabitats inside of a mangrove ecosystem harbor diverse and distinct microbiota, reinforcing the need to conserve these ecosystems as a whole.

## 1. Introduction

Mangroves are coastal ecosystems that have been seriously threatened by anthropogenic activities. Worldwide, mangrove areas have been used for urban development, tourism, oil exploitation, agriculture, and shrimp farming. Between 1980 and 2005, about 3.6 million hectares of mangrove was lost [1]. Competition for land is the major cause of devastation and losses over time. Brazil, the second largest mangrove area in the world, has lost approximately 50,000 ha of mangroves in the last 25 years [2].

Although these ecosystems are well known for their typical flora and associated fauna, comparatively, only a few studies deal with their microbial diversity [3–8]. On the other hand, studies on cultivable microorganisms have advanced in the isolation and identification of organisms capable of degrading xenobiotics, including oil hydrocarbons [3, 5, 9–11].

In the environment microorganisms fulfill various niches and are fundamental for the functioning of mangroves, being particularly important in controlling the geochemistry of these habitats [12, 13]. Recently, using metagenomics and pyrosequencing Andreote et al. [14], Nogueira et al. [15], and Alzubaidy et al. [16] retrieved a large volume of information on the microbial composition and function in tropical mangroves. Although these studies represent a valuable contribution to our understanding of microbial life, more studies are necessary to access the microbial ecology of mangrove sediments from distinct zones inside the mangrove, as well as those submitted to different anthropogenic threats [17].

Genetic fingerprinting techniques provide a pattern or profile of the genetic diversity in a microbial community, which are important in distinguishing PCR products that have different nucleotide sequences. Terminal-Restriction Fragment Length Polymorphism (T-RFLP) has proven to be a valuable tool to study bacterial community structure in complex environments such as sediments and soils [3, 18, 19]. Also, tools for web-based phylogenetic alignment exist that allow the retrieval of hypothetical microbial diversity. These* in silico* methods, such as the resources available on the MiCA3 (Microbial Community Analysis III) website, make the identification of specific organisms in a community based on the length of Terminal-Restriction Fragments (T-RFs) possible, as they predict T-RFs from known and deposited sequences in databases that can be compared with the submitted T-RFs [20, 21].

In this context, we hypothesized that the zonation of mangrove species, as well as the daily fluctuations imposed by the tidal regimes, shapes the microbial communities that are present in these habitats, making them unique to each mangrove area. In order to test our hypothesis, we employed T-RFLP to access the composition and structure of bacterial communities of sediments in vegetated and nonvegetated areas in a mangrove located in Northeastern Brazil and its relation to the biotic and abiotic variables in a region known for petroleum exploitation, which is considered a risk area for oil contamination.

## 2. Materials and Methods

### 2.1. Study Area and Sediment Sampling

**Figure 1:**
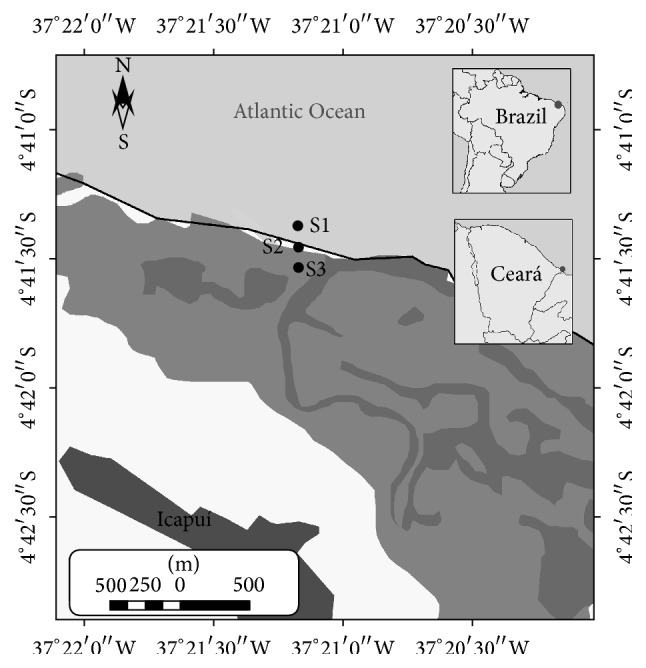
Sampling sites in Barra Grande mangrove, Icapuí, Ceará, Brazil.

Barra Grande mangrove is located in Icapuí, on the extreme east coast of the state of Ceará, Northeastern Brazil (37°20′W 4°40′S), in a region comprised of an extensive tidal flat, covering an area of 1,260.31 ha (Figure 1). Due to the rather flat profile of the studied area, the sampling sites remain uncovered at low tide (0.1 m) and are subsequently flooded by the tide. Sediments from three different sites at depths between 0 and 10 cm were collected, following the shoreline in a perpendicular transect. Site 1 (S1) was the closest to the sea in an area without vegetation; the second site (S2) was located in an area of* Avicennia schaueriana* forest; and the third site (S3) was located in a region of a robust forest of* Rhizophora mangle*. The sites were 150 m apart from each other. At each site, five sediment samples (0–10 cm depth) were randomly collected using a cylindrical sampler (30 cm long and 10 cm in diameter) and transferred to sterile jars. The samples were kept in an ice-cooled box for about 2 hours before being transported to the laboratory. In the laboratory, the five replicate samples from each site were homogenized in order to obtain composed and representative samples of each habitat and a portion was stored at −20°C for DNA extraction and the remaining fraction was used for sediment analyses. Granulometry was performed by dry sieving [22], and organic matter was determined by weight loss on ignition, described in Schulte and Hopkins [23]. The environmental variables pH, salinity, and temperature of the sediments' percolated water were measured directly in the field, using a multiparameter probe (Multiparameter Display System Model 650, YSI, Yellow Springs, OH, USA).

### 2.2. Study of Bacterial Community Structure

Bacterial communities were analyzed by T-RFLP, following the protocol described by Marsh [24]. The extraction of total DNA from sediment samples was performed using the PowerSoil DNA Isolation Kit (Mo Bio Laboratories, Carlsbad, CA, USA), following the manufacturers' protocol. DNA samples were amplified by PCR using the primers 63F labeled with the fluorophore 6-carboxyfluorescein (6-FAM) at the 5′ end and 1389R [25]. PCRs were performed according to the following program: initial denaturation at 94°C for 3 min, 25 cycles of 94°C for 1 min, 55°C for 1 min, 72°C for 2 min, and a final extension at 72°C for 10 min. PCR products were purified using the commercial kit QIAquick PCR Purification (QIAGEN, Valencia, CA, USA). Afterwards, samples were digested separately with restriction enzymes* Hha*I and* Msp*I following the manufacturers' recommendations (New England Biolabs, Beverly, MA, USA). Digestion products were dried at 40°C and sent to the Research Technology Support Facility, Department of Plant Biology, Michigan State University (MSU, East Lansing, MI, USA) Facility, where T-RF profiles were generated. The analysis was performed using 2 *μ*L of the digestion with 8 *μ*L of a solution containing the internal standard MapMaker*™* 1000 (BioVentures Inc., Murfreesboro, TN, USA) labeled with ROX (6-carboxy-X-rhodamine) and the running buffer (deionized formamide). DNA fragments were detected by capillary electrophoresis on an ABI Prism 3100 Genetic Analyzer (Applied Biosystems, Foster City, CA, USA) automatic sequencer. The T-RFs were visualized using the GeneScan Analysis Software (Applied Biosystems), exported to Excel, and analyzed with the Ibest tool (http://mica.ibest.uidaho.edu/) using the height of 50 units of fluorescence as an initial point for the electropherogram analysis and normalized by calculating the relative abundance of each T-RF from the fluorescence intensity area. T-RFs in the range of 50–990 bp were used for the analysis. T-RFs that differed by less than 1 bp were considered identical. The files were exported from Ibest and analyzed by the T-Align tool (http:/inismor.ucd.ie/~talign/index.html). Each individual T-RF was considered an OTU (Operational Taxonomic Unit).

### 2.3. Diversity Indices

The relative abundance of OTUs was used to calculate diversity indices for each sample. The Shannon index (*H*′) by log_2_, the Simpson diversity (*λ*), and the Pielou equitability (*J*′) [26] were calculated using the program Primer 6 (Primer E, Ivybridge, United Kingdom).

### 2.4. Assignment of T-RFs to Bacterial Taxa

The web-based analysis tool (PAT+) provided by MiCA3 (http://mica.ibest.uidaho.edu/pat.php) was used to identify OTUs for T-RF peaks, based on the RDP (Ribosomal Database Project) Release 9.60 16S rRNA gene database [21].

## 3. Results

### 3.1. Characterization of Mangrove Sediments

Habitats S1 and S2 were relatively similar in most environmental factors, except for the presence of vegetation in S2, and salinity was the only analyzed variable shared between S2 and S3, apart from the presence of vegetation. Sediments were classified as fine sand at S1 and S2 and coarse silt at S3, the latter presenting a higher silt + clay and organic matter content. The measured environmental variables temperature, pH, salinity, organic matter content, and sediment particle size from the three habitats of the Barra Grande mangrove are shown in Table 1.

### 3.2. Bacterial Community Structure and Composition

Three different community structures with a higher similarity between S2 and S3 were observed, both inside forested areas. The digestion with* Hha*I generated a larger number of OTUs (120 T-RFs) than* Msp*I (87 T-RFs). This means that* Hha*I best resolved the constituent community in the analyzed samples. Thus, results from the digestion with* Hha*I were selected for further analyses.

**Figure 2:**
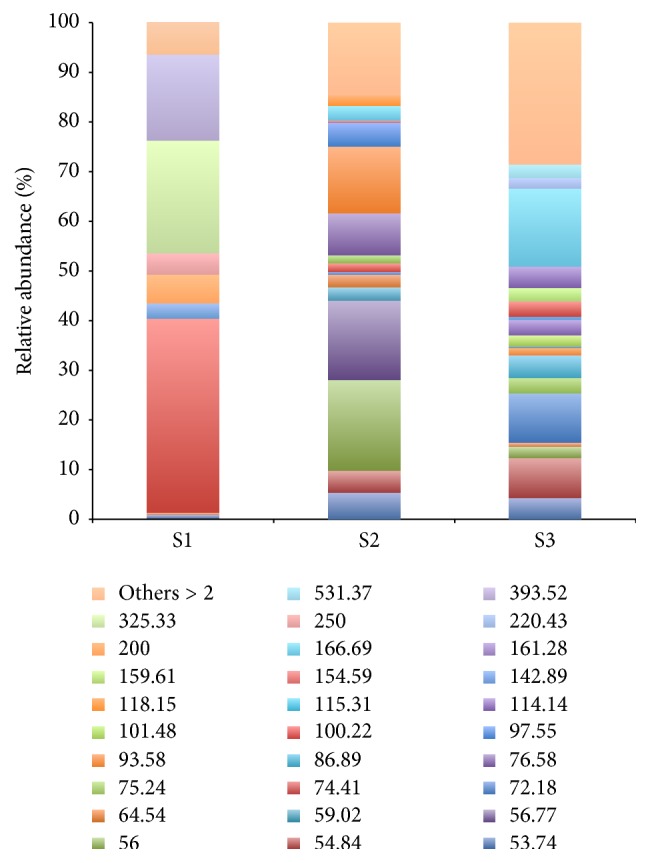
T-RFs and their abundance for bacterial communities of the Brazilian mangrove soils (S1, S2, and S3), derived from* Hha*I digestion. The fragment represented as “others” refers to the sum of all fragments with a relative abundance less than 2%.

**Figure 3:**
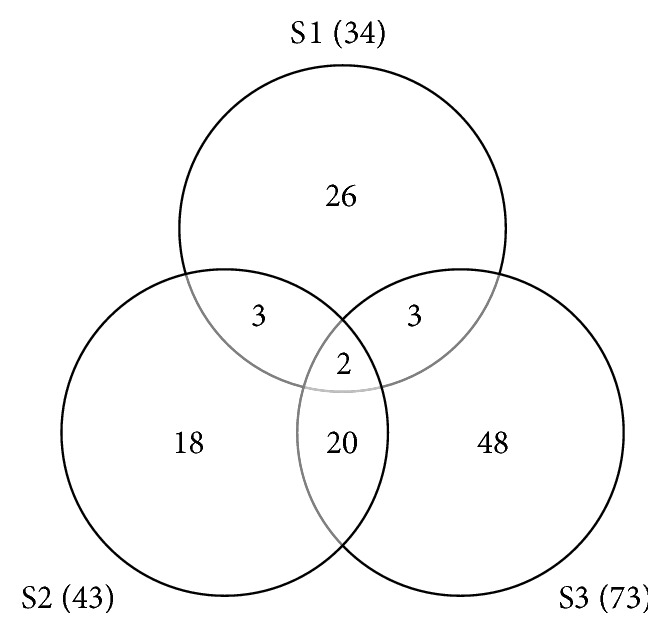
Venn diagrams showing the potential number and shared T-RFs for bacterial communities from Brazilian mangrove soils (S1, S2, and S3) derived from* Hha*I digestion.

The relative abundance of OTUs (Figure 2) revealed that S1 had a lower richness (34 T-RFs) and a great abundance of three OTUs. S3 showed the highest number of T-RFs (73) but a lower relative abundance and was considered the most diverse site in terms of bacterial OTUs. S2 showed intermediate characteristics when compared with the other sites, showing some abundant T-RFs and also an intermediate number of OTUs (43). Only two T-RFs were identical among the sites as shown in Figure 3. In addition, S1 shares only three OTUs with S2 and S3, whereas S2 and S3 share 20 OTUs. Therefore, S1 showed the highest percentage of unique OTUs (76.5%), followed by S3 (65.75%) and S2 (41.86%). The comparison of diversity indices (Table 2) showed an increase in terms of evenness, richness, and diversity from S1 to S3.

Using PAT+ in MiCA, we predicted the potential bacterial groups based on digestion pattern of the fragments obtained by T-RFLP. T-RFs 54 and 65, which were shared by the three sites, were mainly represented by uncultured bacteria of the Bacteroidetes and Proteobacteria phyla. Among the possible species that can be attributed to T-RF 54 are* Flavobacterium* sp.,* Capnocytophaga* sp.,* Vibrio* sp., and* Photobacterium* sp., whereas many species of Bacteroidetes from the Flavobacteriaceae family and some Alphaproteobacteria were assigned to fragment 65.

Considering the dominant fragments at each site, S1 showed three different fragments: T-RF 100 associated with uncultured halophilic bacteria; T-RF 325 represented by uncultured bacteria, including species of Gammaproteobacteria and the cultured bacterium* Vibrio parahaemolyticus*; and T-RF 394 corresponding to an uncultured bacterium. At S2, four dominant fragments were detected: T-RF 57 including Alphaproteobacteria such as uncultured* Azospirillum* sp. and the Bacteroidetes* Mariniflexile fucanivorans* and cultured and uncultured* Cytophaga* spp.; T-RF 56 comprising Alphaproteobacteria as* Sneathiella* sp., uncultured* Mesorhizobium* sp., various species of* Thalassospira*, members of Rhodospirillaceae, some uncultured Gammaproteobacteria from Piscirickettsiaceae, and groups of the phylum Bacteroidetes, such as* Fluviicola* sp.,* Aequorivita* sp.,* A. antarctica*,* A. sublithincola*,* Subsaxibacter* sp.,* Persicivirga* sp.,* Salinimicrobium* sp.,* Mariniflexile gromovii*,* Myroides* sp.,* M. odoratimimus*,* M. profundi*,* M. pelagicus*,* Gelidibacter* sp.,* G. algens*, and an uncultured* Sphingobacterium*; T-RF 77 which could not be identified by the web-based tool; and T-RF 94 which was identified as* Escherichia coli*.

S3 showed three dominant fragments: T-RF 55 represented by uncultured bacteria and* Capnocytophaga* sp.; T-RF 72 represented by uncultured members of the order Bacteroidales; T-RF 167 which consisted of undetermined uncultured bacteria and several uncultured Gammaproteobacteria and cultured representatives such as* Pseudomonas* sp.,* Pseudoalteromonas* sp.,* Shewanella* sp.,* Salicola* sp.,* S. salis*,* S. marasensis*, and* Halovibrio denitrificans*.

## 4. Discussion

In this study, we observed differences in the bacterial community structure and composition that could be attributed to the specific characteristics of each sampled mangrove habitat. It is well known that mangroves are under the influence of marine and terrestrial environments, which generate gradients in the texture of sediments and organic matter content and in salinity as a result of sea and freshwater inputs [27, 28]. Taking this into consideration, it is expected that the fluctuating environmental conditions shape the microbial communities in mangroves.

Peixoto et al., 2011 [8], have shown that mangrove microbial communities are heterogeneously distributed within mangroves and between different mangroves. The authors explain these differences based on the sharp environmental gradients over short spatial scales that include pollutants, reductive-oxidative balance (redox state), pH, and nutrient distribution. The aerobic/anaerobic interface is a critical boundary that characterizes soil community structures.

Also, microbial populations seem to be influenced by the presence and type of mangrove species. Gomes et al., 2010 [29], demonstrated that, even under the fluctuating conditions found in mangroves, the rhizosphere effect, which is well described for terrestrial plants, was also evidenced in this ecosystem. Alzubaidy et al., 2016 [16], found a predominance of Bacteroidetes in the rhizosphere of* Avicennia*, while a predominance of Actinobacteria was evidenced in nonvegetated sediments. Ramírez-Elías et al. [30] studying culturable populations showed that the species in the* Laguncularia* rhizosphere harbored the highest microbial population when compared to other mangrove species.

Sites S2 and S3, which are habitats covered by* A. schaueriana* and* R. mangle*, respectively, shared more similarities in terms of microbial composition with each other than with S1, the site without vegetation. The numbers of OTUs detected in S1, S2, and S3 were 34, 43, and 73, respectively. This increase in the richness from S1 to S3 was observed, which suggests that, besides local abiotic variables, the nature of exudates and nutrients provided by each plant species select specific communities [31].

The observed differences in community structure were reflected in the relative abundance as well as in T-RF composition. Regarding the percentage of unique T-RFs, it is notable that this mangrove harbors quite different bacterial communities, as confirmed by the high value of exclusivity, especially at S1, which was 76.5%, and the low level of similarity among the sites, considering that only two T-RFs were shared by them. Thus, these data confirm that local differences were responsible for distinguishing bacterial populations.

We detected an increase in the evenness of bacterial communities over the three sites; that is, S1 showed lower evenness and the presence of some dominant OTUs. Due to the proximity of the sea, the microbiota in S1 is under the influence of tidal hydrodynamics, which probably led to the selection of several species that are more adapted to marine environments. At S3, a wider distribution of OTUs was observed, with a lower occurrence of dominant OTUs, demonstrating that the environmental conditions have not favored any particular OTU. Intermediate characteristics were shown at S2, which can be explained by its location in an area inside the vegetation (*A. schaueriana*) like S3, but with many abiotic variables similar to those found at S1, due to its proximity to the sea.

Putative community compositions were determined using a phylogenetic assignment tool (PAT) developed by Kent et al. [20], using MiCA3, in which the sizes of T-RF peaks in mangrove soils were matched with T-RF sizes derived* in silico* from the 16S rRNA gene sequences of phylotypes in the RDP database. The results from PAT were used to examine the bacterial community composition at different levels of phylogenetic resolution. At the three studied habitats (S1, S2, and S3), there was prevalence of uncultured bacteria, which shows the wide gap in extant data on microbial diversity, considering the large number of unknown organisms [32].

Taking into account the possible groups associated with T-RFs, it can be observed that, besides the uncultured bacteria, the main common OTUs were phylogenetically affiliated with the Bacteroidetes, with a large number belonging to the Flavobacteriaceae. In Brazilian mangrove sediments, some bacteria affiliated with the Bacteroidetes were also observed in clone libraries, but at a low number compared to the phylum Proteobacteria, which appears to be dominant in these environments [8, 33, 34]. Considering the dominant groups at each habitat in the studied mangrove, an overall abundance of Proteobacteria and Bacteroidetes was observed.

At site S1, which lies in a region close to the sea, without vegetation, there was prevalence of uncultured halophilic bacteria, uncultured Gammaproteobacteria, and* Vibrio parahaemolyticus*. At S2, located in the root zones of* A. schaueriana*, several uncultured bacteria were found, with a predominance of Alphaproteobacteria, some of them known nitrogen fixers, such as an uncultured* Azospirillum*, which was previously reported in roots of* A*.* marina* and in the rhizosphere of* Suaeda monoecious* in a mangrove from India [35]. Several bacterial strains affiliated with the genus* Thalassospira* were detected, which were previously isolated from oil-contaminated seawater. It was found that these could degrade several polycyclic aromatic hydrocarbons (PAHs), including naphthalene, dibenzothiophene, phenanthrene, and fluorene [36]. These strains might play important roles in the bioremediation of marine oil spills and considering the location of the studied mangrove in a risk area for oil contamination, the presence of possible oil degraders indicates the potential of this environment to respond effectively to possible contamination by petroleum hydrocarbons.

Among the OTUs found at S3, some were identified as belonging to the phylum Proteobacteria, such as* Vibrio* sp. and* Photobacterium* (Proteobacteria) and* Rhodovulum sulfidophilum*, a marine photosynthetic bacterium (Alphaproteobacteria). Some members of the genus* Pseudomonas* which are able to metabolize petroleum hydrocarbons [37] were also detected.

Altogether, the data showed distinct bacterial communities among the three mangrove habitats due to the presence and type of vegetation and the divergence environmental variables in which these habitats are submitted. In general, all the potential bacteria corresponding to the identified T-RFs are typical of marine environments and play important roles in maintaining the dynamic balance of the ecosystem. This study highlights the importance of preserving mangrove ecosystems as a whole, due to the uniqueness of each habitat.

## 5. Conclusion

The main contribution of this study was to demonstrate that mangrove soils hold highly diverse bacterial populations with increasing richness from the sea to forested areas, selected by the local differences. The existence of unique bacterial communities to each mangrove habitat covered by distinct plant species clearly demonstrates the importance of the conservation of this ecosystem as a whole.

## Figures and Tables

**Table 1:** Environmental variables of sites S1, S2, and S3 from Barra Grande mangrove soils, state of Ceará, Northeastern Brazil.

Variable	S1	S2	S3
Temperature	31.18 ± 0.4	31.01 ± 0.6	34.6 ± 0.3
pH	9.24 ± 0.25	10.45 ± 0.2	8.8 ± 0.15
Salinity	53.7 ± 0.5	46.5 ± 0.3	46 ± 0.4
Sand (%)	92.0 ± 0.2	93.0 ± 0.15	67.3 ± 0.2
Silt + clay (%)	8.0 ± 0.2	7.0 ± 0.15	32.7 ± 0.2
Organic matter (%)	2.4 ± 0.34	2.7 ± 0.25	8.4 ± 0.6

**Table 2:** Diversity indices generated by T-RFLP profiles of the studied mangrove sites.

Sample	OTUs	*J* ^′a^	*H*′ (log⁡*e*)^b^	*λ* ^c^
S1	34	0.5247	1.85	0.239
S2	43	0.7519	2.828	0.0961
S3	73	0.8096	3.474	0.0555

^a^Pielou's equitability.

^b^Shannon-Weaver's diversity.

^c^Simpson's diversity.
